# Psychosocial factors associated with physical activity in people with dementia: A pilot cross‐sectional study

**DOI:** 10.1002/agm2.12364

**Published:** 2024-11-01

**Authors:** Nicolas Farina, Uzma Niazi, Riona Mc Ardle, Johanna Eronen, Ruth Lowry, Sube Banerjee

**Affiliations:** ^1^ Faculty of Health University of Plymouth Plymouth UK; ^2^ Brighton and Sussex Medical School Brighton UK; ^3^ Translational and Clinical Research Institute Newcastle University Newcastle UK; ^4^ Faculty of Sport and Health Sciences, Gerontology Research Center University of Jyväskylä Finland; ^5^ School of Sport, Rehabilitation and Exercise Sciences University of Essex Colchester UK; ^6^ Faculty of Medicine and Health Sciences University of Nottingham Nottingham UK

**Keywords:** barriers, dementia, exercise, facilitators, motivators, physical activity

## Abstract

**Objectives:**

To understand how psychosocial factors associated with physical activity differ based on disease severity in people with dementia, and how these factors are associated with physical activity participation.

**Methods:**

Eighty‐seven people with dementia, alongside their family carer were asked to complete a series of questions related to physical activity participation, including barriers, motivators, and facilitators. Regression models were developed to understand how psychosocial factors were associated with physical activity participation in the cohort.

**Results:**

In the final models, only the absence of intrapersonal barriers was associated with overall physical activity and regular moderate‐to‐vigorous physical activity. Feelings of relatedness were associated with regular moderate‐to‐vigorous physical activity only.

**Conclusion:**

Reducing intrapersonal barriers would appear to be a potentially useful strategy to promote physical activity in people with dementia. However, a tailored approach is needed depending on the desired physical activity outcome.

## Graphical abstract

Factors significantly associated with physical activity outcomes in people with dementia in the final regression models. The figure highlights that psychosocial factors are associated with different constructs related to physical activity. Only the presence of intrapersonal barriers to physical activity was associated with both total physical activity and regular moderate‐to‐vigorous physical activity.

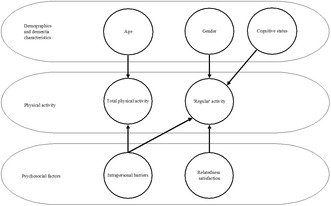

## 1 INTRODUCTION

The number of people living with dementia is on the rise, with an estimated 50 million people with dementia worldwide, which is expected to reach 152.8 million by 2050.^1^ Dementia is defined, in part, by progressive decline of cognition and function. Dementia directly or indirectly results in poorer health outcomes that ultimately shape a person's quality of life.

For people with dementia, physical activity has a number of reported psychological and health benefits,^2^, ^3^ including helping with neuropsychiatric symptoms.^4^ Physical activity has been reported to slow cognitive decline,^5^, ^6^, ^7^ though the evidence of its therapeutic value on cognition is still mixed, with robust randomized controlled trials not finding benefit,^8^ Physical activity for people with dementia may have the added benefit of reducing carer burden and stress.^9^, ^10^, ^11^

UK government guidelines recommend 150 min of moderate physical activity or 75 min of vigorous physical activity per week, or equivalent in bouts of at least 10 min at a time.^12^ These guidelines recognize that physical activity can be difficult for people with functional impairment, such as people with dementia, and thus even small doses of activity are better than being entirely sedentary.^13^ Evidence from the Health Survey for England reinforces the decline in activity with age, while the majority of adults adhere to the guidelines, activity reduces considerably in old age (i.e., 69% adherence in 19–64 year olds vs. 50% adherence in those aged 65+).^14^

People with dementia are less active than cognitively healthy older adults,^15^ participating in fewer sporting activities,^16^ and frequently adopting low‐intensity activities such as walking^17^ and gardening.^18^ In part, this can be attributed to a general decline in activities as a result of functional and cognitive impairment, leading to a “shrinking world.”^19^ It is therefore unsurprising that there is often an emphasis of quantitative research to understand how disease‐related factors (e.g., cognitive decline) or contexts (e.g., care home residence) are associated with physical activity participation.^20^, ^21^ Efforts to form a more comprehensive picture of mechanisms underlying physical activity engagement in dementia are limited. Much of the literature is composed of small‐scale qualitative research.^22^ This can limit the generalizability of findings and prevents us from quantifying the size and strength of associations. As such, we are often left with a large number of reported barriers and motivators,^22^, ^23^ but little understanding how they cluster and are empirically associated with physical activity engagement.

Conceptually, in the literature concerning healthy adults there are an abundance of models of physical activity participation. These models have been applied to the design of interventions and public health messages to increase engagement and efficacy. Theories such as self determination theory (SDT)^24^ provide us with insights into the importance of why individuals pursue specific goals and behaviors such as physical activity. Other models, such as the social‐ecological model provide a multidimensional framework, highlighting that behavior is influenced by variables at an individual level but also at a broader social and society level.^25^ The variation and significance of different behavioral models of physical activity has been described elsewhere.^26^ Efforts to consolidate these models for people with dementia have been developed^27^, ^28^ in which the importance of significant others (e.g., carers) features prominently. Despite this, there are very few studies that have used these behavioral models when trying to understand barriers, motivators, and facilitators of physical activity in people with dementia.

In this pilot study, we present the first quantitative data highlighting the psychosocial barriers, motivators, and facilitators surrounding physical activity in people with dementia. The research seeks to provide insights into how these psychosocial factors differ based on disease severity, so we can better understand whether they change as the disease progresses. We also aim to identify which factors have the greatest effect on physical activity participation in people with dementia.

## 2 METHODS

This methodology for the host research project that this is part of is described elsewhere.^29^ Not reported here, a subset of this cohort also participated in qualitative interviews that explored the barriers, motivators, and facilitators to physical activity in people with dementia and their carers.^30^

### 2.1 Participants

Participants were recruited from South East England as a sub study of the MODEM research program.^31^ Participants were included if they were diagnosed with dementia (any dementia subtype, with no restriction on other co‐morbidities) and had a family carer who was able and willing to report on the physical habits of the person with dementia. Participants were identified through lists of individuals who had previously expressed interest in research, clinical referral from local memory assessment services, self‐referral through Join Dementia Research (http://joindementiaresearch.nihr.ac.uk/), or self‐referral through community groups. The recruitment strategy encouraged a range of dementia severities (i.e., we approached care homes for people with severe dementia).

### 2.2 Procedure

Participants (the person with dementia) and informants (family carer) were visited in their homes (or another location if requested). Both the person with dementia and their carer were informed about the study and were assessed for eligibility. If the potential participant met the inclusion criteria, they were asked to provide informed consent before participation. Capacity to consent was formally assessed for all people with dementia. To assess capacity, the researcher talked through the study and checked whether the participant: (a) understood the purpose of the study, (b) was able to retain information long enough to make a decision, (c) weighed up the information to make a decision, and (d) communicated their decision. If the person lacked capacity to consent, a family member or friend were identified to act as a personal consultee. Measures related to subjective psychosocial elements (e.g., attitudes, perceived barriers) were self‐completed by the person with dementia, whereas the measures of physical activity participation were completed by the carer as an informant report. Visits lasted approximately 90 min.

### 2.3 Measures

- *Demographic information*—age, gender, ethnicity, dementia diagnosis (subjective), highest education level, and employment (current, or if unemployed, previous employment).
- *Standardized Mini‐Mental State Examination* (*sMMSE*)^32^—a 12 component measure of cognitive impairment. Score range from 0 to 30; lower scores represent greater cognitive impairment.
- *Behavioral Regulation in Exercise Questionnaire* (*BREQ‐3*)^33^—A reliable and valid measure for quantifying behavior regulation for exercise described in the self‐determination theory. This 24‐item measure includes the subscales: amotivation, external regulation, introjected regulation, identified regulation, integration regulation, and intrinsic motivation. Each subscale is a mean of each itemset, with higher scores (max = 4) representing greater alignment with the subdomain. The relative autonomy index (RAI) was calculated to provide a single index of the extent to which participants feel self‐determined in exercise, with positive scores representing greater relative autonomy and negative scores indicated more controlled regulation.
- The Psychological Need Satisfaction in Exercise Scale (PNSE)^34^—A 18‐item measure of perceived satisfaction of their basic psychological needs of exercise, namely, competence (i.e., self‐belief that they can perform exercise), autonomy (i.e., freedom to choose and participate in the exercise), and relatedness (i.e., feeling connected to others). Each sub‐scale consists of six items that are totaled and a mean score calculated.
- Older Persons' Attitudes to Physical Activity and Exercise Questionnaire (OPAPAEQ)^35^—A 14‐item measure of four physical activity attitudes namely tension relief, promotion of health, vigorous exercise, and social benefits. The responses on the five‐point Likert scale are totaled for each subscale and a mean score calculated.
- *Barriers to Outdoor Physical Activity Questionnaire* (*BOPAQ*)^36^—A 17‐item measure of barriers to outdoor physical activity, each item had a yes/no response. An additional single item was included to capture any other barriers not captured in the questionnaire. Novel to this study, items were conceptually grouped into intrapersonal barriers, interpersonal barriers and community barriers (See supplementary material A—Data S1).
- *The Community Healthy Activities Model Program for Seniors* (*CHAMPS*) *physical activities questionnaire for older adults* ^37^—The questionnaire estimates weekly frequency and duration of meaningful physical activities. CHAMPS lists activities of various intensities from light to vigorous.
- *The Rapid Assessment of Physical Activity* (*RAPA*)^38^—*aerobic subscale* is a seven‐item questionnaire that captures progressively more frequent and intense aerobic physical activity patterns.
- *Carer co‐participation –* Following each item within the CHAMPS, informants were asked how often they co‐participated in the activity with the person with dementia (1 = Never, 2 = Rarely, 3 = Occasionally, 4 = A moderate amount, 5 = a great deal). The mean score was calculated across valid items.

### 2.4 Analysis

To understand how physical activity and psychosocial factors were affected by dementia severity, participants were initially split by severity (sMMSE 10–19 = moderate severity, sMMSE ≥20 = mild severity). Hot Deck Imputation^39^ was applied to cases (*n =* 6) where there were few missing items (≤10%). Due to high levels of missing self‐report data, people with severe dementia (MMSE <10, *n =* 37) were excluded from the analysis. An additional 10 participants were excluded because they were missing large amounts of sMMSE data or another key demographic variable.

Regression models were created for each outcome, to understand differences between people with mild dementia and moderately severe dementia, after adjusting for age and gender. Multi‐stage multiple regression models were used to understand the factors associated with physical activity participation, as measured by total physical activity per week (CHAMPS) and regular moderate to vigorous physical activity (RAPA). Linear and logistic regression models were used, respectively. In the first stage, a series of regression models were created in which age, gender (1 = male, 0 = female) and sMMSE score were controlled for. In the final stage, age, gender, and sMMSE were controlled for, alongside all variables that were statistically significant (*p* < 0.05) in first stage of the model.

Regression coefficients were reported alongside 95% bias corrected and accelerated bootstrapped (1000 resamples) Confidence Intervals.

### 2.5 Ethics

Ethical approvals were obtained from the National Social Care Research Ethics Committee (17/IEC08/0042).

## 3 RESULTS

Eighty‐seven people with mild (*n =* 53, 61%) and moderately (*n =* 34, 39%) severe dementia participated. Forty‐seven participants (56%) had an Alzheimer's disease diagnosis. Participants were on average 78.0 years old (SD = 8.59), and predominantly male (*n =* 60, 69%). The most frequently reported highest level of education was O‐level or equivalent (*n =* 28, 32.2%), with 13 participants (*n =* 14.9%) having no formal education, and 19 participants (20.8%) completing a degree or post‐graduate education. All people with dementia were White British, White Irish, or White other (*n =* 87, 100.0%). Thirty‐one participants (35.6%) were either working, or had previously worked, in a level 4 skilled job (Standard Occupational Classification 2020). The informants within the study were either a spouse or long‐term partner (*n =* 73, 84%), or son or daughter (*n =* 14, 16%). Participants on average participated in over 13 h per week of physical activity, and 25 (29%) reported that they were regularly active.

### 3.1 Dementia severity

Participants with moderately severe dementia participated in 4.7 fewer hours physical activity per week (CHAMPS) compared to those with mild‐severity dementia, after adjusting for age and gender (*p* = 0.036). People with moderately severe dementia also participated in less regular moderate to vigorous physical activity compared to the mild‐severity dementia group, as measured by the RAPA (*p* = 0.043). See Table 1.

**TABLE 1:** Descriptive statistics of psychosocial outcomes and physical activity participation, split by mild severity (sMMSE >20, *n =* 53) and moderate severity (sMMSE 10–19, *n =* 34) dementia.

	Total		Mild		Moderate		Difference	
	*M* (SD)	*N* (%)	*M* (SD)	*N* (%)	*M* (SD)	*N* (%)	Co‐ef (95% BCa)^a^	*p*‐value
Motivation								
Amotivation (0.0 to 4.0), *n =* 80	0.37 (0.75)		0.26 (0.57)		0.58 (0.97)		0.208 (−0.162 to 0.541)	0.283
External (0.0 to 4.0), *n =* 80	0.88 (1.12)		0.75 (1.11)		1.13 (1.13)		0.410 (−0.138 to 0.958)	0.140
Introjected (0.0 to 4.0), *n =* 80	1.40 (1.16)		1.35 (1.17)		1.48 (1.15)		0.134 (−0.381 to 0.699)	0.634
Identified (0.5 to 4.0), *n =* 80	3.11 (0.97)		3.13 (0.84)		3.10 (0.95)		0.078 (−0.356 to 0.517)	0.708
Integrated (0.0 to 4.0), *n =* 80	2.74 (1.33)		2.83 (1.26)		2.59 (1.47)		−0.108 (−0.806 to 0.654)	0.746
Intrinsic (0.0 to 4.0), *n =* 80	3.08 (1.10)		3.19 (1.05)		2.86 (1.18)		−0.270 (−0.765 to 0.287)	0.308
RAI (−11.6 to 24.0), *n =* 80	13.55 (8.07)		14.73 (7.73)		11.36 (8.37)		−2.525 (−6.408 to 1.280)	0.209
Satisfaction								
PSNE Competence (1.0 to 6.0), *n =* 67	3.97 (1.51)		4.16 (1.38)		3.54 (1.74)		−0.308 (−1.275 to 0.576)	0.576
PSNE Autonomy (1.0 to 6.0), *n =* 69	5.59 (0.80)		5.68 (0.59)		5.41 (1.11)		−0.316 (−0.923 to 0.178)	0.279
PSNE Relatedness (1.5 to 6.0), *n =* 48	5.35 (1.04)		5.46 (0.80)		5.16 (1.39)		−0.168 (−1001 to 0.493)	0.629
Attitudes								
Tension (2.0 to 5.0), *n =* 81	4.00 (0.66)		4.02 (0.61)		3.96 (0.74)		−0.026 (−0.365 to 0.326)	0.887
Health (2. 7 to 5.0), *n =* 81	4.28 (0.53)		4.34 (0.52)		4.18 (0.54)		−0.153 (−0.410 to 0.090)	0.256
Social (2.0 to 5.0), *n =* 81	4.08 (0.66)		4.09 (0.71)		4.07 (0.59)		−0.016 (−0.296 to 0.288)	0.930
Vigorous (1.5 to 5.0), *n =* 79	3.39 (0.79)		3.38 (0.80)		3.43 (0.77)		0.083 (−0.302 to 0.468)	0.669
Barriers								
Interpersonal barriers (Yes), *n =* 85		4 (4.6%)		1 (1.9%)		3 (9.4%)	1.767 (−15.233 to 19.736)	0.016
Intrapersonal barriers (Yes), *n =* 85		41 (48.2%)		19 (35.8%)		22 (68.8%)	1.461 (0.323 to 2.99)	0.004
Community barriers (Yes), *n =* 85		62 (72.9%)		39 (73.6%)		23 (73.9%)	−0.257 (−1.434 to 0.924)	0.619
No barriers (Yes), *n =* 84		14 (16.5%)		9 (17.0%)		5 (15.6%)	0.152 (−1.636 to 1.557)	0.807
Facilitators								
Mean co‐participation frequency (1.0 to 5.0), *n =* 81		2.77 (1.25)		2.54 (1.17)		3.17 (1.30)	0.678 (0.051 to 1.218)	0.024
Physical activity								
CHAMPS: All physical activity (hours/week) (0.00–53.75) *n =* 87	13.08 (11.34)		15.75 (11.52)		8.91 (9.83)		−4.687 (−9.052 to −0.3298)	0.036
RAPA: aerobic regular active (Yes), *n =* 87		25 (28.7%)		20 (37.7%)		5 (14.7%)	−1.093 (−2.544 to −0.038)	0.043

*Note*: Outcome names include range (in parenthesis), and number of valid cases of the total sample.

^a^ Adjusted for age and gender.

Those with moderately severe dementia were four times more likely to have at least one intrapersonal barrier (OR = 4.311, 95% CIs 1.614 to 11.516), and nearly six times more likely to have at least one interpersonal barrier (OR = 5.853, 95% CIs 0.546 to 62.783). Weaker associations were observed between dementia severity and the presence of community barriers (OR = 0.774, 95% CIs 0.276 to 2.165) and the absence of barriers (OR = 1.164, 95% CIs 0.333 to 4.070). Carer co‐participation was higher for those with moderately severe dementia (B = 0.678, BCa 95% CIs 0.051 to 1.218).

All other indices (i.e., attitudes, behavioral regulation and satisfaction) did not significantly differ between severity groups (*p*‐values >0.05), see Table 1.

### 3.2 Psychosocial factors associated with physical activity

Two barriers (interpersonal and intrapersonal barriers) and two measures of satisfaction (competence and autonomy) were associated with total physical activity participation after controlling for age, gender, and cognitive status. In addition, more intrinsic motivation was associated with more physical activity participation. No measure of exercise attitudes, or carer co‐participation were associated with overall physical activity participation (*p*‐values >0.05; see Table 2). Statistically significant associations were brought forward into a final model, alongside age, gender, and cognitive status. In the final model, younger age, absence of intrapersonal barriers, and feelings of competence were most strongly associated with physical activity participation. See Table 3 for further details.

**TABLE 2:** Regression model of psychosocial factors association with measures of physical activity participation.

	CHAMPS: Total time physically active (hours/week)	*p*‐value	RAPA: Regularly active (yes)	p
	B (95% CIs BCa)^a^		B (95% CIs BCa)^a^	
Satisfaction				
Competence	**2.880 (1.432 to 4.753)**	**0.003**	0.467 (−0.057 to 1.480)	0.057
Autonomy	**3.133 (0.866 to 6.259)**	**0.012**	0.634 (−0.440 to 2.251)	0.136
Relatedness	2.552 (0.169 to 6.937)	0.059	**1.141 (−0.039 to 7.909)**	**0.029**
Barriers				
Intrapersonal	**−8.003 (−11.744 to −4.446)**	**0.001**	**−2.259 (−3.640 to −1.623)**	**0.002**
Interpersonal	**−9.185 (−17.711 to −1.490)**	**0.012**	**−20.382 (−21.621 to −18.497)**	**0.001**
Community barriers	−4.964 (−10.698 to 1.228)	0.086	0.095 (−1.082 to 1.375)	0.867
No barriers	5.317 (−2.024 to 12.166)	0.141	0.231 (−1.544 to 1.678)	0.720
Attitudes				
Tension	0.215 (−3.886 to 5.260)	0.925	−0.314 (−1.265 to 1.014)	0.490
Health	2.143 (−1.937 to 7.716)	0.340	0.583 (−0.741 to 2.450)	0.376
Social	2.442 (−0.919 to 5.946)	0.167	0.371 (−0.622 to 1.558)	0.381
Vigorous	0.319 (−2.893 to 3.701)	0.853	−0.119 (−0.847 to 0.672)	0.728
Motivation				
RAI	**0.410 (0.201 to 0.620)**	**0.003**	0.050 (−0.039 to 0.176)	0.127
Carer co‐participation				
Average frequency of co‐participation	0.957 (−1.565 to 3.849)	0.429	0.018 (−0.424 to 0.441)	0.933

*Note*: Bold reflects statistically significant associations.

Abbreviation: BCa, Bias corrected and accelerated.

^a^ Adjusted for age, gender, and sMMSE score.

**TABLE 3:** Statistically significant variables from model 2 entered CHAMPS (hours/week).

	B	LCI	UCI	Standardized B	*p*‐value	Adjusted *R* ^2^
	31.182					0.273
Age	−0.442	−0.736	−0.190	−0.32	0.011	
Gender	0.410	−4.850	6.244	0.02	0.881	
sMMSE	0.200	−0.347	1.011	0.10	0.512	
Motivation: RAI	0.059	−0.249	0.353	0.04	0.655	
Intrapersonal barriers	−6.163	−10.884	−1.748	−0.27	0.014	
Interpersonal barriers	−1.345	−15.033	16.253	−0.02	0.836	
Competence	1.658	−0.035	3.367	0.21	0.070	
Autonomy	1.286	−2.380	6.275	0.09	0.373	

*Note*: Confidence intervals are bias corrected and accelerated (based on 951 samples).

Comparatively, intrapersonal and interpersonal barriers, and feelings of relatedness were associated with being regularly active, as measured by the RAPA, see Table 2. In the final model, feelings of relatedness, and the absence of intrapersonal and interpersonal barriers were associated with being regularly active. Notably, the wide confidence intervals for interpersonal barriers indicate less precision in the estimate. In the model being male and higher cognitive status were associated with regular activity. Unlike the CHAMPs model, age was not associated with regular activity, see Table 4 for further details.

**TABLE 4:** Statistically significant variables from model 2 entered simulation—RAPA (1 = Regularly active).

	B	LCI	UCI	OR	*p*‐value	Nagelkerke *R* ^2^
Constant	−4.569					0.669
Age	−0.125	−12.443	−0.05	0.883	0.153	
Gender	−4.254	−21.316	−7.89	0.014	0.003	
sMMSE	0.386	‐	‐	1.471	0.018	
Relatedness	1.859	−925.620	599.528	6.417	0.010	
Intrapersonal barriers	−5.474	−25.113	−5.197	0.004	0.007	
Interpersonal barriers	−6.684	−13.022	24703.537	0.001	0.657	

*Note*: Confidence intervals are bias corrected and accelerated (based on 856 samples).

## 4 DISCUSSION

Our research is the first study to report the empirical associations between psychosocial variables and physical activity in people with dementia, drawing from behavioral models. The findings highlight that physical activity participation was lower in people with moderately severe dementia compared to those with mild dementia, and this coincides with the increased likelihood of intrapersonal and interpersonal barriers. The presence of intrapersonal barriers was an important determinant of physical activity participation in people with dementia.

At the sample level, dementia severity was associated with less physical activity participation, reflecting previously reported severity group differences.^40^ It is important to recognize that these differences should not be interpreted as cognitive impairment leading to less physical activity; previous literature suggests that cognitive function is not consistently associated with physical activity levels in people with dementia.^40^, ^41^, ^42^ In fact, in our final model, cognitive status was not associated with total physical activity participation.

Barriers to outdoor physical activity were common, with the majority reporting a community barrier (73% e.g., slippery paths), and nearly half reporting an intrapersonal barrier (48%; e.g., fatigue). Very few participants reported an interpersonal barrier (5%; e.g., no one to go with). Such findings are important, as those who participated in the qualitative interviews in this cohort did not report community or interpersonal barriers, but did report intrapersonal barriers (e.g., cognitive impairment and poor physical health).^30^ As such, the mere presence of barriers tells us little about its importance to the participants' own habits. Another consideration is the limited items within the interpersonal barriers domain (*n =* 2) do not capture the true breadth of interpersonal barriers or might miss nuances in interpersonal relationships. For example, the person with dementia might feel that they have someone to go with, but that person might still shape physical activity participation.

Moderate dementia severity coincided with an increased prevalence of intrapersonal and interpersonal barriers, but not community barriers, which remained high in both mild‐ and moderate‐severity dementia. Carer co‐participation in physical activity was common amongst the physical activities performed, though on average co‐participation occurred “rarely” or “occasionally.” Carer co‐participation occurred more frequently in those with moderately severe dementia. These findings, support the notion that as impairment increases, so does the time needed to support people with dementia to perform these activities.^43^ No other factor commonly associated with physical activity participation (i.e., motivation, attitudes and satisfaction of physical activity) differed between severity groups. Participants tended to be intrinsically motivated to participate in physical activity, and there was a shift toward extrinsic regulation in the moderate‐severity group. Such a finding is perhaps surprising considering that apathy increases with severity,^44^ although we need to be vigilant that self‐reported motivation for physical activity may deviate from broader informant‐reported apathy.

The presence of intrapersonal and interpersonal barriers was negatively associated with physical activity participation after adjusting for age, gender, and cognitive status. On average, the presence of these barriers was associated with 8 and 9 h less physical activity per week, respectively. In addition, greater perceived satisfaction of exercise was associated with more frequent participation. Competence, autonomy, and relatedness were all positively associated with physical activity as measured by the CHAMPS. Within SDT, each of these components are considered an important basic human need, and thus is an important motivator for behavior.^24^ As such, people exist on a continuum of self‐determination that extends from the high levels being intrinsically motivated to lower levels reflecting external regulation and at the extreme, amotivation. In line with this, we observe that people with dementia who identify as being intrinsically motivated are more likely to participate in physical activity. Previous evidence indicates that carers play an important role in motivating and facilitating physical activity^45^ which we also observed in our qualitative substudy.^30^ As such, we might hypothesize that carer co‐participation could indicate greater extrinsic regulation of behavior. However, there was no such association in our model. Our findings do not preclude the possibility that carers do influence physical activity participation,^28^, ^46^ albeit the association may be more complex than captured here or was masked by observation bias that occurs through using informant report measures.^47^

In the final model, which incorporated all statistically significant factors from the previous stage, we identified that the presence of intrapersonal barriers was the only factor, outside of age, strongly associated with physical activity participation. Importantly, the model indicated that the presence of intrapersonal barriers leads to, on average, 6.2 fewer physical activity hours per week. Intrapersonal factors such as fatigue and health‐related restrictions have been reported as common barriers for older adults,^48^ and have also been reported in people with dementia.^30^, ^49^ Intrapersonal barriers are commonly ranked as the most important barriers, motivators, and facilitators for people for dementia.^50^ Our findings therefore indicate that cognitive impairment does not primarily drive overall physical activity, but rather, it is associated age‐related and health‐related barriers that are likely to occur alongside dementia progression. All previous associations retained the same direction of effect in the final model (e.g., the presence of an interpersonal barrier was associated with 1.1 fewer hours per week).

Notably, when developing the model for a more discrete threshold of “regularly active,” we saw similarities and differences when compared to total physical activity participation. In terms of similarities, intrapersonal and interpersonal barriers were associated with being regularly active. Intrinsic motivation to be physically active was not associated with being regularly active, and neither was perceived need fulfillment of autonomy. Instead, the basic psychological needs of relatedness (*p* < 0.05) were associated with being regularly active. In the final model, we found that being male, higher cognitive performance, relatedness satisfaction and fewer intrapersonal barriers were associated with being regularly active. Although relatedness is an important component of SDT, it should be noted that its associations are not always observed because exercise can occur in solitude.^51^ As such, our findings indicate that people with dementia who are regularly active do not do it alone. We therefore suggest that mechanisms to be physically active in people with dementia vary depending on the type of physical activity participated in. The fact that feelings of relatedness featured within the model, could indicate that those who are regularly active do so because they are able to interact with others.

Differences between the final models of the two outcomes can make interpretation difficult. Assuming that differences are based on intensity differences, rather than measurement error, it means that different strategies are needed to achieve a specific frequency of moderate‐to‐vigorous intensity physical activity. If we adopt the view that something is better than nothing, then tackling intrapersonal barriers should be the priority. Whereas, if regular moderate‐to‐vigorous physical activity is the desired outcome, then we need to consider how we can promote feelings of relatedness in the physical activities. Feelings of relatedness can be achieved by showing empathy, warmth, value, and respect.^52^ Although not conceptualized in terms of relatedness, social support is flagged as a promising feature to promote physical activity in people with dementia.^23^, ^53^ Peers have previously been used as means to promote relatedness in exercise interventions in older adults,^54^, ^55^ whilst also helping to overcome barriers.^56^

There are several important limitations of this study to consider. First, there are variations in terms used in the outcome measures. For example, the measures of barriers emphasized outdoor physical activity, whilst the satisfaction measure focused on exercise. Conceptually, such terms have subtle differences, which could bias responses provided. Second, the cognitive impairment of the person with dementia may influence accurate recall. To minimize such bias, elements that we saw as being more episodic (e.g., frequency of physical activity participation) were answered by the informant, rather than the person with dementia. Adopting such an approach, whilst common practice in research, does mean that observation bias may occur for informant reported outcomes. Third, due to the cross‐sectional nature of the research, we are unable to be certain about the direction of effect or potentially reciprocity. For example, in previous cross‐sectional research the relationship between cognition and physical activity have either inferred^57^ or explicitly concluded that physical activity benefits cognitive function directly.^58^ Fourth, the relatively small sample size of our study increases the possibility of type II error, whilst multiple comparisons may increase type I error. In a regression model with eight variables (power = 0.8, *p*‐value = 0.05) we would need 108 participants to detect a medium effect size. Fifth, our findings should not be assumed to be generalizable outside of the characteristics of the underlying cohort, for example our sample is composed of exclusively of White participants. Finally, our study did not seek to replicate theoretical models such as the PHYT‐in‐dementia model (Physical Activity Behavior change Theoretical model in dementia),^28^ but we can potentially observe that certain constructs (e.g., personal characteristics, support) may be less important in naturalistic observations.

## 5 CONCLUSIONS

Increased cognitive impairment does not necessarily lead to less physical activity overall. Differences between severity groups are likely to be explained, at least partially, by the presence of intrapersonal barriers in people with moderately severe dementia. Importantly, motivation to be physically active, perceived satisfaction and attitudes toward physical activity remain unchanged between severity groups. Tackling perceived intrapersonal barriers appears to be a priority if we want to increase physical activity participation overall in people with dementia. Although further research is needed to replicate these findings in a larger sample, we should recognize that different strategies maybe needed to promote regular moderate‐to‐vigorous physical activity in people with dementia.

## AUTHOR CONTRIBUTIONS

Nicolas Farina: Conceptualization, methodology, formal analysis, writing—review and editing, writing—original draft, project administration, supervision, data curation, Uzma Niazi: Conceptualization, writing—original draft. Riona Mc Ardle: Writing—review and editing. Johanna Eronen—Resources, writing—review and editing. Ruth Lowry: Writing—review and editing. Sube Banerjee: Writing—review and editing, supervision, funding acquisition.

## FUNDING INFORMATION

The MODEM study is funded by the UK Economic and Social Research Council (ESRC) and the National Institute for Health Research (NIHR) (ES/L001896/1). Beyond approval for our original proposal, neither funding body has had any influence over the design of MODEM, the collection, analysis, or interpretation of data, or the writing of this manuscript. Dr. Farina and his independent research are supported by the National Institute for Health Research Applied Research Collaboration South West Peninsula. The views expressed in this publication are those of the author(s) and not necessarily those of the National Institute for Health Research or the Department of Health and Social Care. Dr. McArdle is funded by the National Institute for Health and Care Research (NIHR) for her fellowship (NIHR 301677), and supported by the NIHR Newcastle Biomedical Research Centre (BRC) based at The Newcastle upon Tyne Hospital NHS Foundation Trust, Newcastle University and the Cumbria, Northumberland and Tyne and Wear (CNTW) NHS Foundation Trust.

## CONFLICT OF INTEREST STATEMENT

None to declare.

## ETHICS STATEMENT

Ethical approvals were obtained from the National Social Care Research Ethics Committee (17/IEC08/0042).

## Supporting information


Data S1.

## Data Availability

The data generated and analyzed during the current study are not publicly available due to consent not being obtained for this purpose but are available from the corresponding author on reasonable request.
