# Supplementary material for: Psychosocial factors associated with physical activity in people with dementia: A pilot cross‐sectional study
Source: Aging Med (Milton). 2024 Nov 1;7(5):543–52. doi: 10.1002/agm2.12364 (PMC11535167; doi:10.1002/agm2.12364)
Supplement: Supplementary file 1 — Data S1. [file AGM2-7-543-s001.docx]

| Supplementary file A: Grouping of barriers and their occurrence within the sample | | | |
| --- | --- | --- | --- |
|  |  | Responded Yes | |
|  | Missing | N | % |
| Intrapersonal (at least 1) | 2 | 41 | 48.2% |
| Pain | 2 | 22 | 25.9% |
| Energy | 3 | 17 | 19.5% |
| Vision | 2 | 7 | 8.2% |
| Hearing | 2 | 8 | 9.2% |
| Fear falling | 2 | 9 | 10.3% |
| Insecure | 3 | 11 | 12.6% |
| Not interested | 3 | 4 | 4.6% |
| Too old | 3 | 6 | 6.9% |
| Not used to it | 3 | 9 | 10.7% |
| Interpersonal (at least 1) | 2 | 4 | 4.7% |
| No one to go with | 2 | 3 | 3.4% |
| Told not to go | 4 | 1 | 1.2% |
| Community (at least 1) | 2 | 62 | 72.9% |
| Crime | 3 | 6 | 7.1% |
| Car | 5 | 9 | 10.3% |
| Darkness | 2 | 26 | 29.9% |
| Weather | 3 | 41 | 47.1% |
| Slippery | 4 | 39 | 47.0% |
| Environment | 4 | 6 | 6.9% |
| Other | 3 | 16 | 19.0% |
